# Comment on Higher Post‐Operative L‐Thyroxine Dosage Needed After Right Compared to Left‐Lobe Hemithyroidectomy

**DOI:** 10.1002/edm2.70362

**Published:** 2026-10-13

**Authors:** Nayab Ikram, Madeeha Amir

**Affiliations:** ^1^ University of Health Sciences Lahore Pakistan; ^2^ Khyber Medical College Peshawar Pakistan

To the Editor,

1

We have read with interest the study by Deischinger et al. [1] investigating the association between the side of hemithyroidectomy and postoperative levothyroxine requirements. It provides comparative evidence between right‐ and left‐ sided hemithyroidectomy and suggests that right‐ sided hemithyroidectomy may be associated with higher postoperative levothyroxine requirement. Unlike other studies focusing on patient‐related factors, this study also considers surgical site and we commend the authors for this contribution. We would like to raise points that deserve further consideration.

First, postoperative TSH was higher following right‐sided surgery than left‐sided surgery (1.70 vs. 1.40 μIU/mL, *p* = 0.011), yet the model adjusted for preoperative rather than postoperative TSH. Thus, the groups were compared at different postoperative biochemical levels. Additionally, a dose recorded at a single visit, without documented titration, may not accurately reflect a stable dose requirement [2, 3]. A sensitivity analysis restricted to patients with TSH within the target range would help determine whether the observed effect persists after accounting for postoperative biochemical differences.

Secondly, thyroid volume measurements were available in only 112/426 patients (26%). The authors suggested thyroid volume resection as a main reason for the higher levothyroxine requirement [1], but thyroid volume assessment was performed in only a limited number of patients. Previous evidence has also linked thyroid remnant volume with post‐operative hypothyroidism [4]. So, it limits the dependence of higher levothyroxine on resected thyroid volume. Future studies should obtain standardized thyroid volume measurements in the full cohort.

Third, the adjusted difference (+7.9ug/day; 95% Cl, 1.38–14.45) is smaller than a typical levothyroxine titration increment of 12.5–25 μg [2], while the observed correlation was weak (*r*_s_ = −0.103) [1]. The clinical significance of this relatively small difference for postoperative management therefore remains uncertain.

We raised these points not to diminish authors' work, but to highlight that future studies should assess LT4 requirements after stable biochemical optimization, with TSH and LT4 measurements alongside thyroid‐volume assessment. This may help clarify whether the observed higher LT4 dose after right‐sided hemithyroidectomy represents a true side‐related requirement and strengthen the evidence for this association.

## Author Contributions

**Nayab Ikram:** writing – review and editing, writing – original draft, resources, project administration, visualization, funding acquisition, supervision, formal analysis, validation, investigation, software, data curation, methodology, conceptualization. **Madeeha Amir:** writing – review and editing.

## Funding

The authors have nothing to report.

## Ethics Statement

The authors have nothing to report.

## Consent

The authors have nothing to report.

## Conflicts of Interest

The authors declare no conflicts of interest.

## Data Availability

Data sharing not applicable to this article as no datasets were generated or analysed during the current study.
